# Supplementary material for: Assembling highly repetitive Xanthomonas TALomes using Oxford Nanopore sequencing
Source: BMC Genomics. 2023 Mar 27;24:151. doi: 10.1186/s12864-023-09228-1 (PMC10045945; doi:10.1186/s12864-023-09228-1)
Supplement: Supplementary file 1 — Additional file 1. PDF file integrating Supplementary Figs. S1 – S9. [file 12864_2023_9228_MOESM1_ESM.pdf]

Supplementary Figures for Assembling highly repetitive Xanthomonas TALomes using Oxford Nanopore sequencing

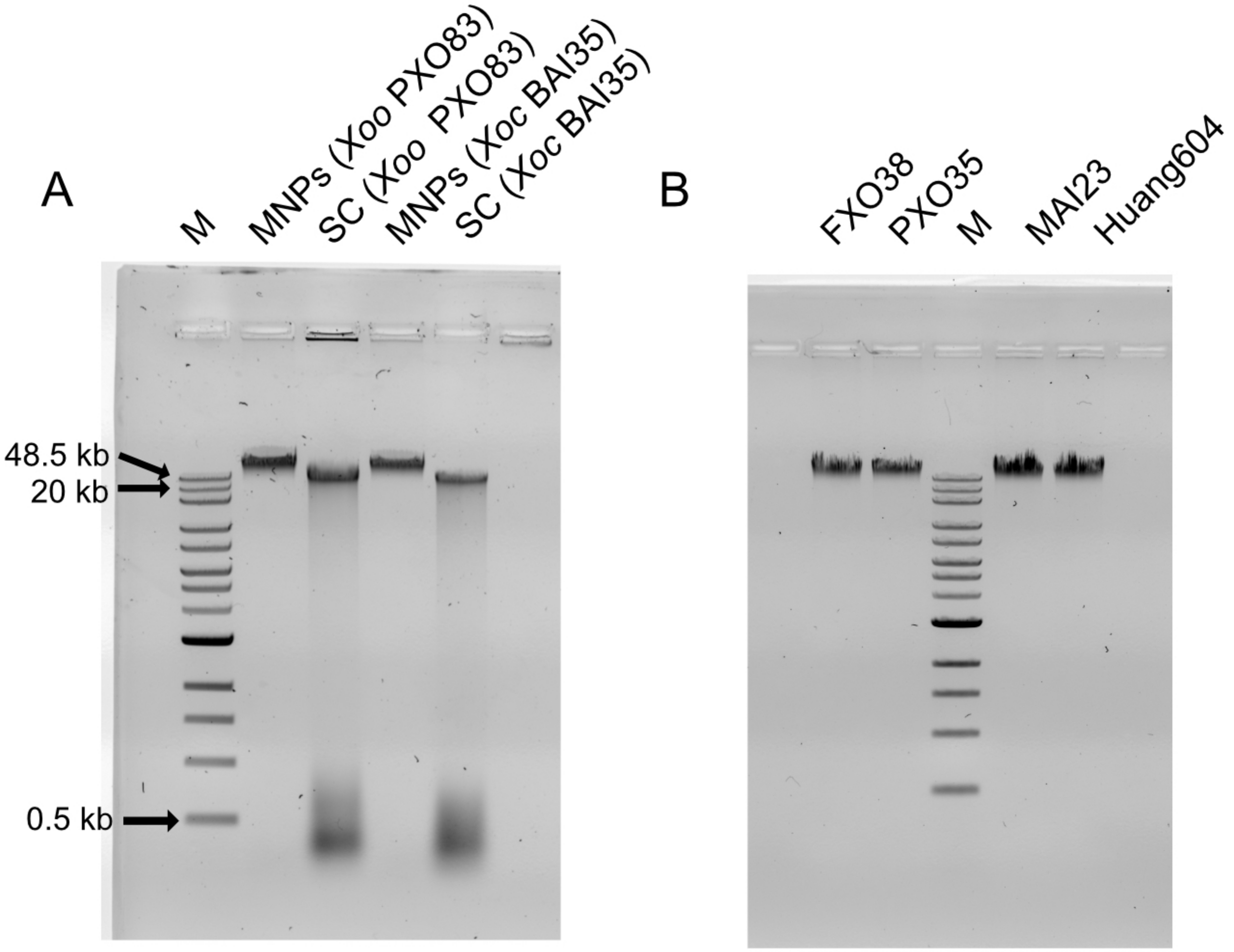

Supplementary Figure S1: Quality control of genomic DNA. Agarose gel electrophoresis (0.5% agarose) of gDNA from (A) *Xanthomonas oryzae* pv. *oryzae* strain PXO83 and *Xanthomonas oryzae* pv. *oryzicola* strain BAI35 purified with magnetic nano particles (MNPs) or spin column (SC) and (B) *Xanthomonas oryzae* strains used in this study. Lane M, 1 kb extend DNA size marker (NEB).

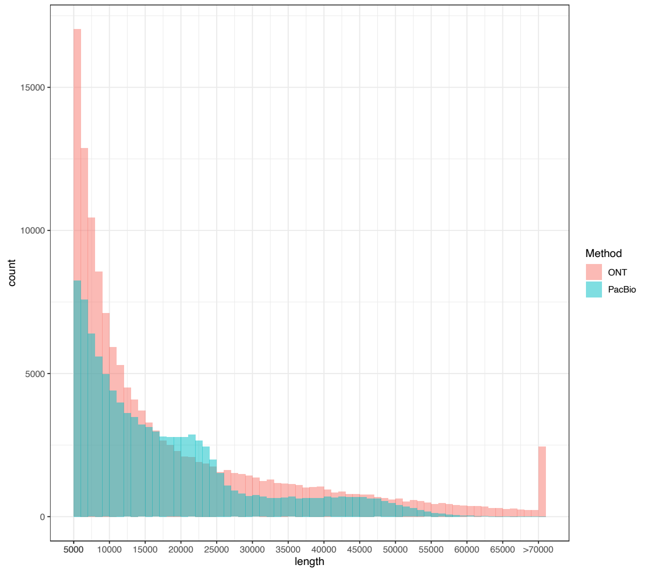

Supplementary Figure S2: Histograms of the read lengths above 5 kbp in PacBio and ONT libraries for *Xoo* PXO35. Read lengths above 70 kbp are aggregated in a single bin. A number of $45,582$ PacBio reads and $439,861$ ONT reads have lengths below 5 kbp.

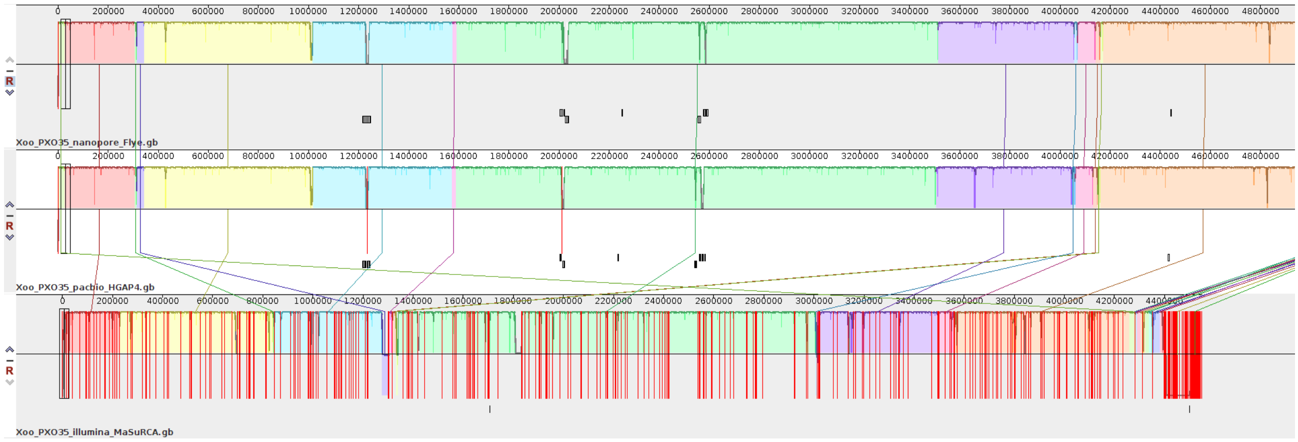

Supplementary Figure S3: Genomic alignment of ONT-based, PacBio-based and Illumina-based PXO35 assemblies using progressiveMauve for a subset of the assemblies shown in Figure **??**. Contig borders are marked by red vertical lines. Large TALE clusters in the ONT-based assembly are located at contig borders of the PacBio-based assembly.

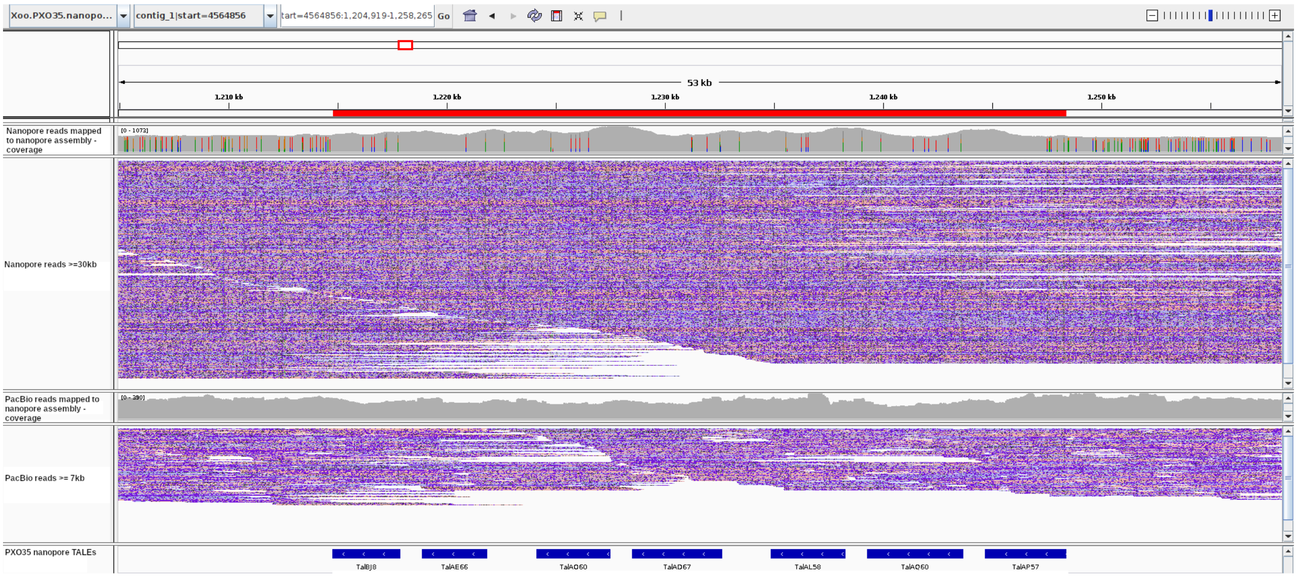

Supplementary Figure S4: IGV screenshot of the ONT-based, computationally corrected assembly of *Xoo* PXO35 in the region of a large TALE cluster at approx. 1,220 kbp. The first coverage track refers to all ONT reads that map to the region of the cluster. The corresponding alignment track contains only long ONT reads with a length of at least 30 kbp. The second coverage track refers to all mapped PacBio reads and the corresponding alignment track only includes PacBio reads with a length of at least 7 kbp. Mapped reads are sorted according to start location. The large TALE cluster reconstructed from the ONT reads using Flye is well-supported by ONT but also by PacBio reads. A number of 85 ONT reads spans this cluster completely (cf. Supplementary Table **??**), whereas none of the PacBio reads spans the complete cluster despite the continuous coverage with PacBio reads.

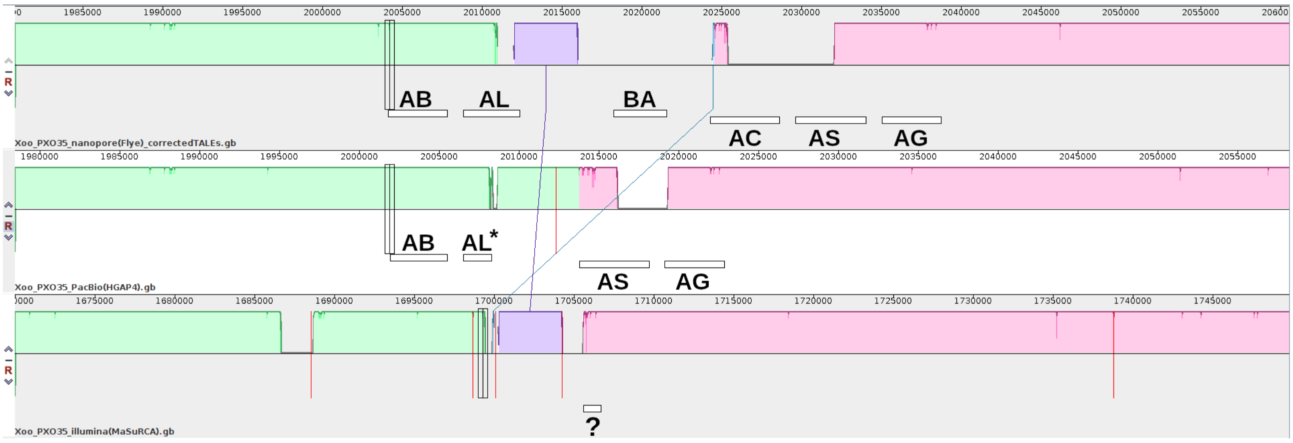

Supplementary Figure S5: A large cluster of repetitive TALE genes in *Xoo* PXO35 at approx. 2,000 kbp prevents a contiguous assembly. The asterisk indicates a truncated TALE gene. Contig borders are marked by red vertical lines. The large TALE cluster cannot be completely resolved from PacBio reads, while TALEs are missing in the Illumina-based assembly.

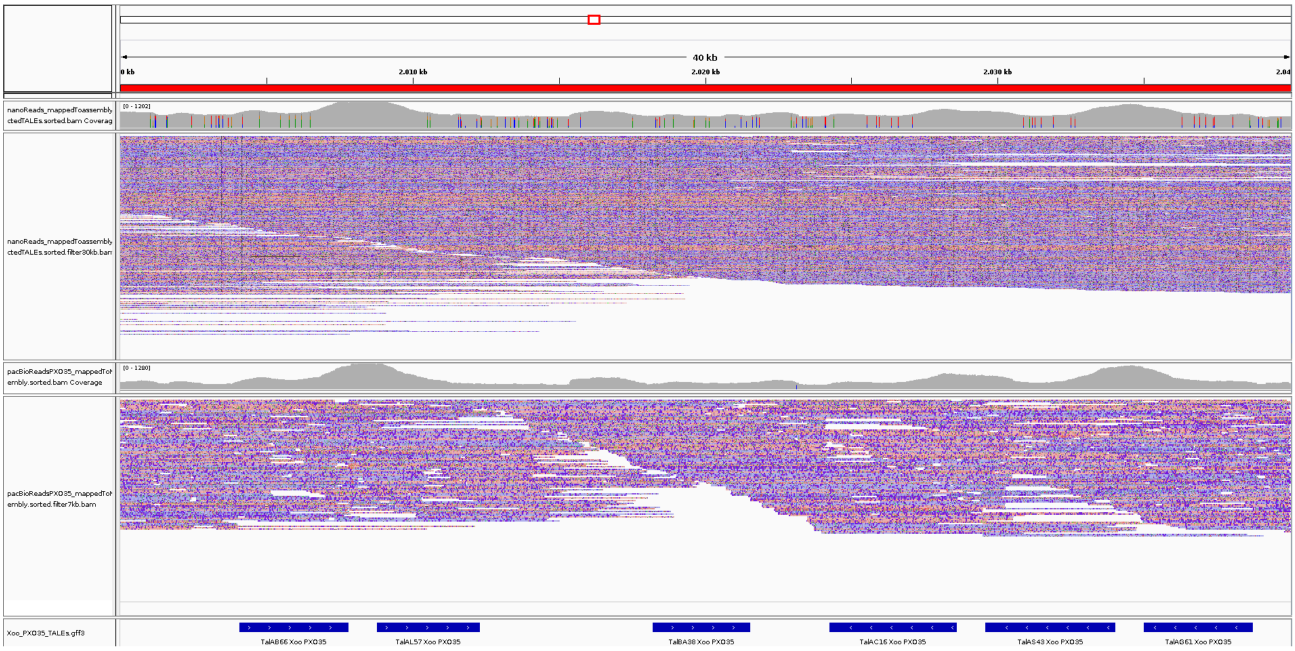

Supplementary Figure S6: IGV screenshot of the ONT-based, computationally corrected assembly of *Xoo* PXO35 in the region of a large TALE cluster at approx. 2,000 kbp. The first coverage track refers to all ONT reads that map to the region of the cluster. The corresponding alignment track contains only long ONT reads with a length of at least 30 kbp. The second coverage track refers to all mapped PacBio reads and the corresponding alignment track only includes PacBio reads with a length of at least 7 kbp. Mapped reads are sorted according to start location. A number of 47 ONT reads spans this cluster completely (cf. Supplementary Table **??**), whereas none of the PacBio reads spans the complete cluster despite the continuous coverage with PacBio reads.

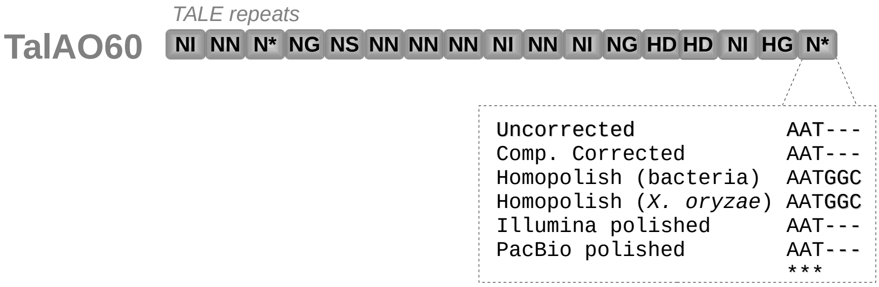

Supplementary Figure S7: Comparison of the polishing result of Homopolish using 'bacteria' or *X. oryzae* as a reference with the uncorrected assembly, the computationally corrected assembly, the assembly polished using Illumina reads, and the assembly using PacBio reads for TalAO60 of *Xoo* PXO35. We find that in both variants, Homopolish introduces a codon for an additional amino acid, which is not supported by the polishing variants based on experimental data (Illumina/PacBio).

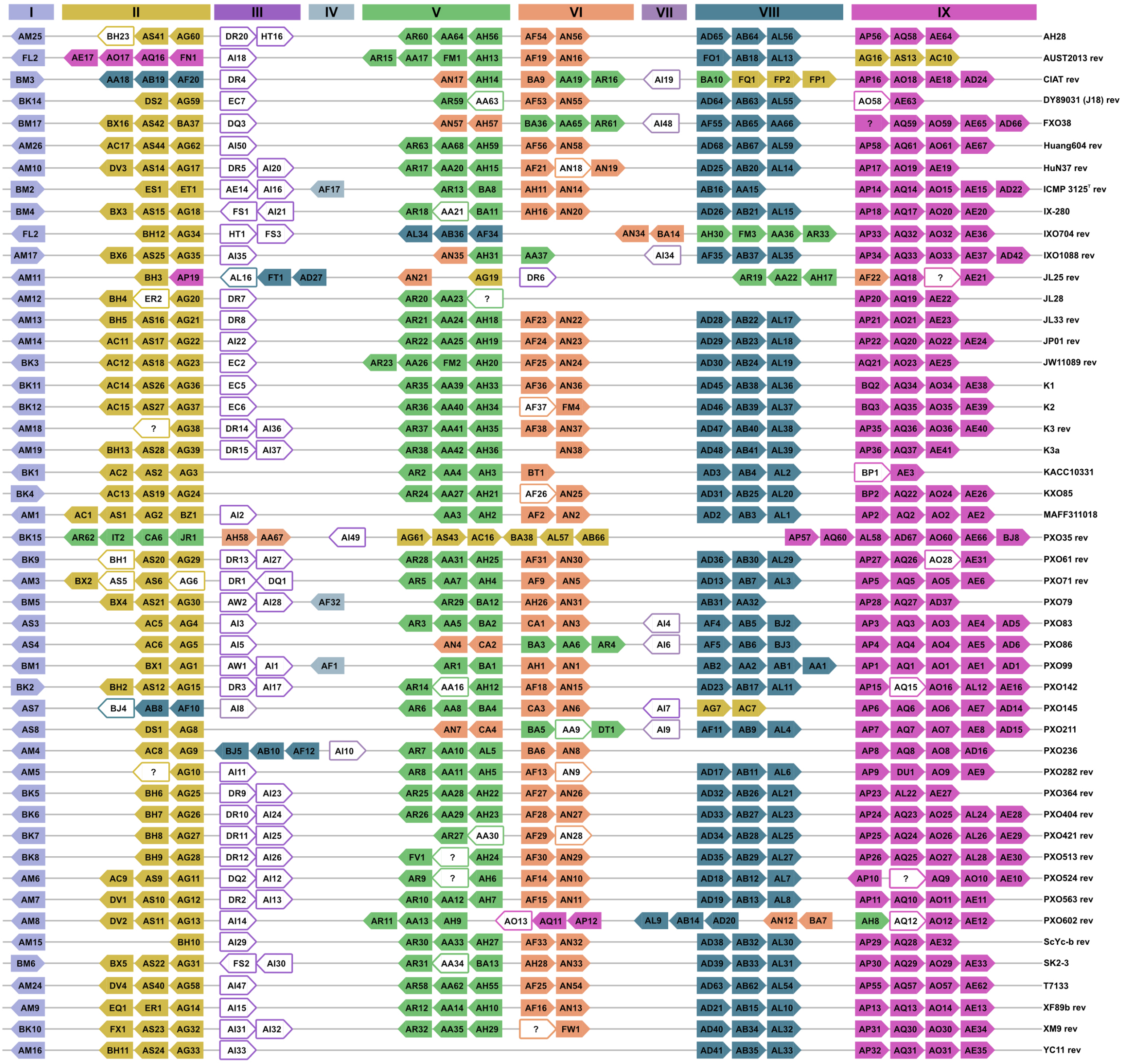

Supplementary Figure S8: Overview of TALE cluster assignment of sequenced Asian *Xoo* strains. TALEs are represented as arrows, directions indicate the relative orientation in the corresponding genome. All TALEs are assigned to classes using AnnoTALE and named accordingly. The two capital letters of the TALE class is shown and in addition an allele number which is unique for every particular TALE gene and assigned to distinguish TALEs from different bacterial strains. TALE clusters are defined at the top, affiliations are represented by colors. Strain names are shown at the right. Pseudo TALE genes are represented by a colored outlined white arrow. If a TALE could not be assigned to a class by AnnoTALE, the arrow is marked with a question mark. Strains with large genomic rearrangements may be shown reverse complemented for clarity, which is indicated by "rev" after the strain name.

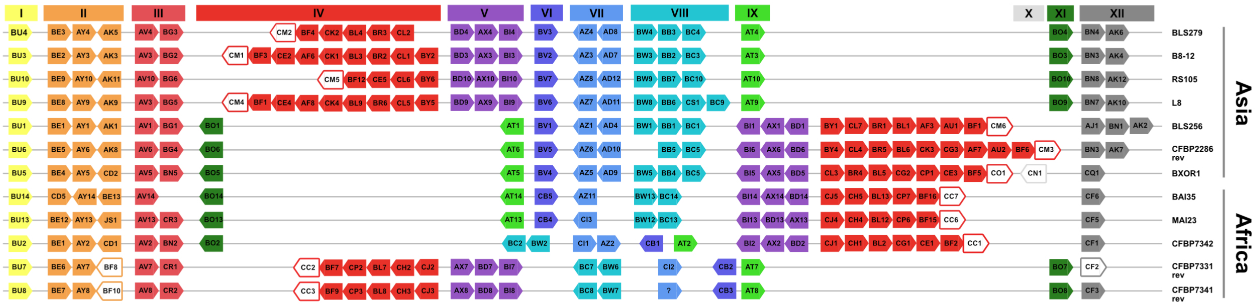

Supplementary Figure S9: Overview of TALE cluster assignment of sequenced Asian and African *Xoc* strains. TALEs are represented as arrows, directions indicate the relative orientation in the corresponding genome. All TALEs are assigned into classes using AnnoTALE and named accordingly. TALE clusters are defined at the top, affiliations are represented by colors. Strain names and geographical origin are shown at the right. Pseudo TALE genes are represented by a colored outlined white arrow. If a TALE could not be assigned to a class by AnnoTALE, the arrow is marked with a question mark. Strains with large genomic rearrangements are shown reverse complemented for clarity, which is indicated by "rev" after the strain name.
