# Supplementary material for: Assembling highly repetitive Xanthomonas TALomes using Oxford Nanopore sequencing
Source: BMC Genomics. 2023 Mar 27;24:151. doi: 10.1186/s12864-023-09228-1 (PMC10045945; doi:10.1186/s12864-023-09228-1)
Supplement: Supplementary file 2 — Additional file 2. PDF file integrating Supplementary Tables A, B, C, E. [file 12864_2023_9228_MOESM2_ESM.pdf]

Supplementary Tables for Assembling highly repetitive Xanthomonas TALomes using Oxford Nanopore sequencing

Supplementary Table A: Statistics of read lengths in the PacBio and ONT library for *Xoo* PXO35.

Read length	# PacBio reads	# ONT reads
< 1000	$7,468$	$252,612$
> 10000	$65,952$	$84,042$
> 20000	$32,796$	$46,786$
> 30000	$15,005$	$29,486$
> 40000	$8,317$	$17,932$
> 50000	$1,907$	$10,257$
> 60000	78	$5,338$
> 70000	1	$2,447$

Supplementary Table B: Number of differences, InDels and Mismatches (MM), within TALE genes between the ONT-based assembly after computational correction and the hybrid ONT + Illumina (Unicycler) assembly, separately for the N-terminal (N-term), C-terminal (C-term) and repeat region (repeats) of each TALE. Compared sequences are determined by blastn of the TALE DNA sequences of the computationally corrected assembly against the genomic sequence according to the hybrid assembly.

TALE	InDels(N-term)	InDels(repeats)	InDels(C-term)	MM(N-term)	MM(repeats)	MM(C-term)
TalAB66	9	2	6	0	0	0
TalAL57	9	5	4	0	0	1
TalBA38	8	6	3	1	0	0
TalAI49	3	16	1	0	1	0
TalJR1	5	0	8	0	0	1
TalCA6	12	8	3	0	0	0
TalIT2	7	4	6	0	0	0
TalAR62	11	8	2	0	0	0
TalBK15	8	7	5	2	0	0
TalAH58	8	8	1	0	9	1
TalAA67	13	14	3	0	1	0
TalAG61	8	0	4	0	0	0
TalAS43	7	0	2	0	3	0
TalAC16	8	1	4	0	0	0
TalAP57	7	4	6	0	0	1
TalAQ60	7	24	1	1	2	0
TalAL58	12	4	4	0	2	0
TalAD67	6	1	2	0	0	0
TalAO60	6	4	5	0	0	0
TalAE66	9	3	2	0	0	0
TalBJ8	8	3	8	0	0	0

Supplementary Table C: Overview of the effect of Homopolish on annotated TALE genes in the *Xoo* PXO35 assembly. For each TALE that has been annotated after computational correction, we indicate (+/-) if the TALE is also present in the AnnoTALE annotation after polishing with Homopolish using 'bacteria' or *X. oryzae* as reference. Missing TALEs are the result of premature stops or frame shifts introduced by Homopolish within the TALE sequence. In addition, we list differences in RVDs introduced by Homopolish, which typically lead to the introduction of an additional, 13th amino acid in the repeat (e.g., N* → NG), or the deletion of the 13th amino acid (e.g., NN → N*). One specific example of such an introduction of a 13th amino acid is illustrated in Supplementary Figure S7. In some cases (TalAA, TalJR), the modifications introduced by Homopolish lead to a different class assignment of the corresponding TALE.

PXO35 TALEs (comp. corrected)	Homopolish (bacteria)	RVD differences	Homopolish (X. oryzae)	RVD differences
TalAA	+	-	TalJT	HG → H*
TalAB	+	-	+	-
TalAC	-		+	-
TalAD	+	-	+	-
TalAE	+	-	+	-
TalAG	+	-	+	-
TalAH	+	N* → NG	+	N* → NG
TalAI	+	-	+	-
TalAL	+	-	+	-
TalAO	+	N* → NG	+	N* → NG
TalAP	+	-	+	-
TalAQ	+	-	+	-
TalAR	+	-	+	Last 3 RVDs missing (truncated)
TalAS	+	N* → NG	+	-
TalBA	-		-	
TalBJ	+	N* → ND	+	N* → NG
TalBK	+	-	+	-
TalCA	+	HG → H*, NN → N* , N* → HN	-	
TalIT	+	-	+	-
TalJR	TalJS	N*→NG, N*→ HN	TalJS	N* → NG, N* → NG

Supplementary Table E: N50 read length of sub-samples of fractions of the complete set of ONT reads.

Fraction	read N50
100.00%	23,888
75.00%	23,927
50.00%	24,030
25.00%	24,112
12.50%	24,059
6.25%	24,024
3.12%	24,173
