# Supplementary material for: Assembling highly repetitive Xanthomonas TALomes using Oxford Nanopore sequencing
Source: BMC Genomics. 2023 Mar 27;24:151. doi: 10.1186/s12864-023-09228-1 (PMC10045945; doi:10.1186/s12864-023-09228-1)
Supplement: Supplementary file 4 — Additional file 4. Preparation of Beads Solution. [file 12864_2023_9228_MOESM4_ESM.pdf]

Supplementary Methods for Assembling highly repetitive Xanthomonas TALomes using Oxford Nanopore sequencing

Preparation of Beads Solution

Unwashed MNPs were vortexed thoroughly for 5 min. The required amount of beads (e.g. 200 µl for 2 ml of beads solution) was transferred to a 2.0 ml reaction tube, 1 ml of ddH2O was added and the mixture thoroughly vortexed. The tube was then placed on a magnetic rack. After 1 min, the supernatant was removed, and another 1 ml of ddH2O was added. This was repeated 3 times, or until the supernatant becomes clear after all MNPs were stuck to the tube wall when placed on a magnetic rack. To prepare the beads solution (10 mM Tris-HCl, pH 8.0; 1mM EDTA; 1.6 M NaCl; 11 % PEG 8000 (w/v); 0.2 % Tween 20 (v/v); 10 % washed MNP´s (v/v)), all compounds, without the washed MNPs and the PEG were mixed in a 1.5 ml tube. 1 ml of the premixed solution was used to resuspend the washed MNPs and transfer them to the 1.5 ml tube. In the end, the PEG was added, using a cut 1000 µl pipet-tip. All compounds were autoclaved as stock solutions before used.
